# The effect of opioids on gastrointestinal function in the ICU

**DOI:** 10.1186/s13054-021-03793-1

**Published:** 2021-10-24

**Authors:** Yun Yan, Yu Chen, Xijing Zhang

**Affiliations:** 1 Department of Anaesthesiology and Perioperative Medicine, Xijing Hospital, The Fourth Military Medical University, Xi’an, China; 2 Department of Critical Care Medicine, Xijing Hospital, The Fourth Military Medical University, Xi’an, China

**Keywords:** Opioids, Critical illness, Gastrointestinal function, Gastrointestinal microbiome

## Abstract

Gastrointestinal (GI) dysfunction is common in the critical care setting and is highly associated with clinical outcomes. Opioids increase the risk for GI dysfunction and are frequently prescribed to reduce pain in critically ill patients. However, the role of opioids in GI function remains uncertain in the ICU. This review aims to describe the effect of opioids on GI motility, their potential risk of increasing infection and the treatment of GI dysmotility with opioid antagonists in the ICU setting.

## Introduction

A round-table conference in 1997 concluded that GI function was crucial for the clinical outcomes of ICU patients and was associated with poor prognosis [1]. However, there is no unanimity about the definition of GI dysfunction [2–4]. The Working Group on Abdominal Problems (WGAP) recommended the term “acute gastrointestinal injury” (AGI) with four grades of severity be used to describe the GI function of critically ill patients [5]. AGI severity grades are better associated with clinical outcomes in critically ill patients and predict prognosis [6, 7]. GI problems are common and occur in 50–60% of ICU patients [8]. A total of 59.1% of patients had at least one GI symptom during their first ICU stay, and 36.2% had two or more symptoms [9]. GI dysfunction includes high gastric residuals, gastroesophageal reflux, aspiration, constipation, diarrhoea, and abdominal distention and is associated with a prolonged length of stay in the ICU and increased mortality [10–13]. In this review, we provide an update on the impact of opioids on gastrointestinal function.

Pain is ubiquitous in the ICU, with 50% of ICU patients suffering from moderate to severe pain [14]. Pain in the ICU has multiple aetiologies, including underlying illness, invasive therapy, incisions, daily care, penetrating catheters and tubes [15]. Furthermore, delirium, impaired communication, sleep deprivation and preexisting chronic pain exacerbate the pain experienced in the ICU [15]. Opioids are the cornerstone treatment for moderate to severe pain. Opioid prescriptions have quickly skyrocketed, and they are commonly administered in the ICU, with 63–86% of ICU patients treated with opioids [16, 17]. However, the adverse effects of opioid therapy cause discomfort, seriously impact the patient’s quality of life and can even lead to the discontinuation of treatment. Studies have shown that opioids strongly inhibit the GI tract [18–21]. The GI tract is sensitive to low doses of opioids, and an animal study demonstrated that one-quarter of the morphine needed to produce analgesia inhibits intestinal motility and that one-twentieth of the analgesic dose is enough to treat diarrhoea [22].

### Distribution and physiological function of opioid receptors in the GI tract

**Fig. 1:**
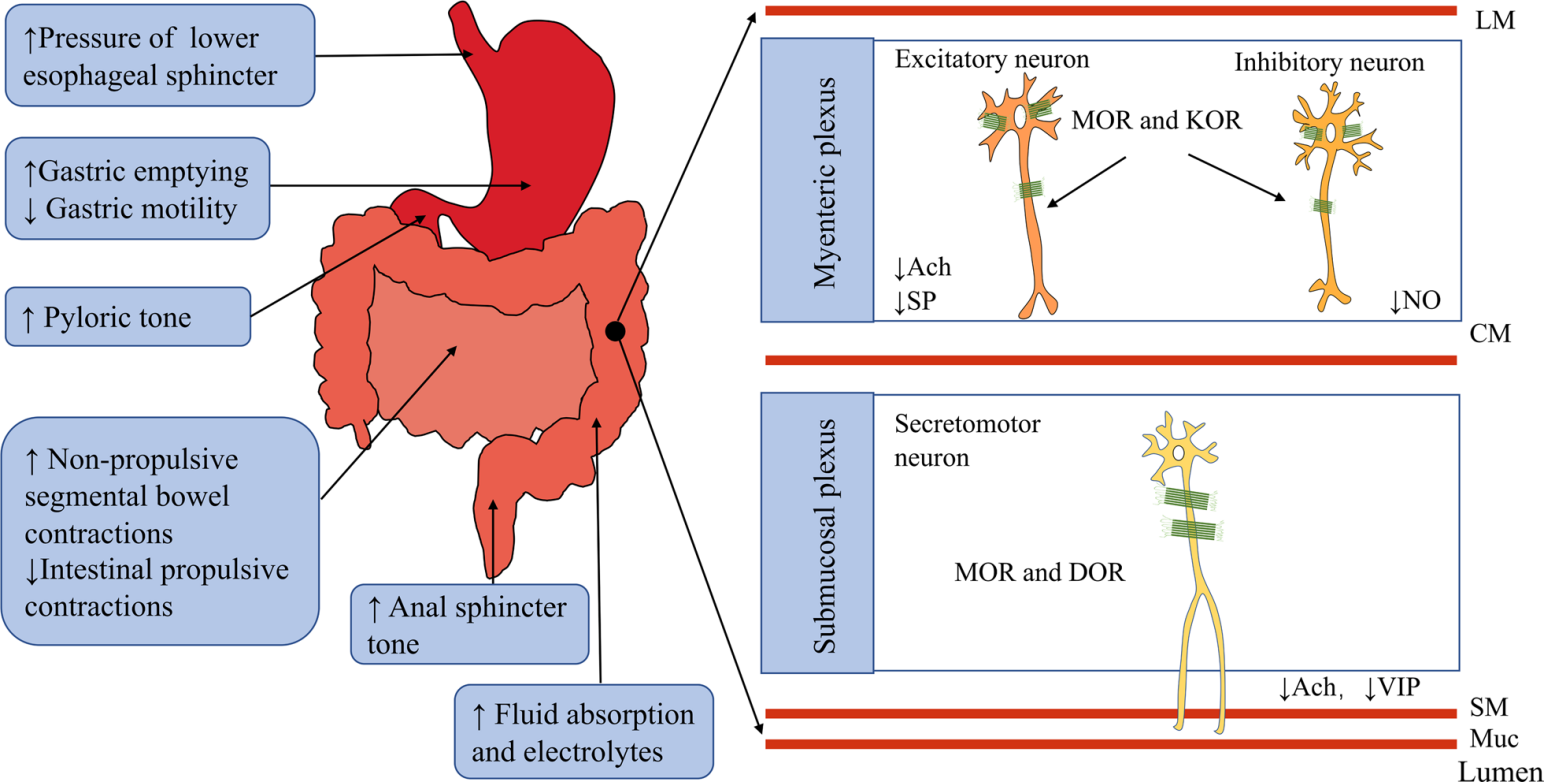
Summary of opioid-induced effects in the GI system. MOR mu opioid receptor, DOR delta opioid receptor, KOR kappa opioid receptor, CM circular muscle, LM longitudinal muscle, SM submucosa, Muc mucosa, Ach acetylcholine, SP substance-P, NO nitric oxide, VIP vasoactive intestinal polypeptide

**Fig. 2:**
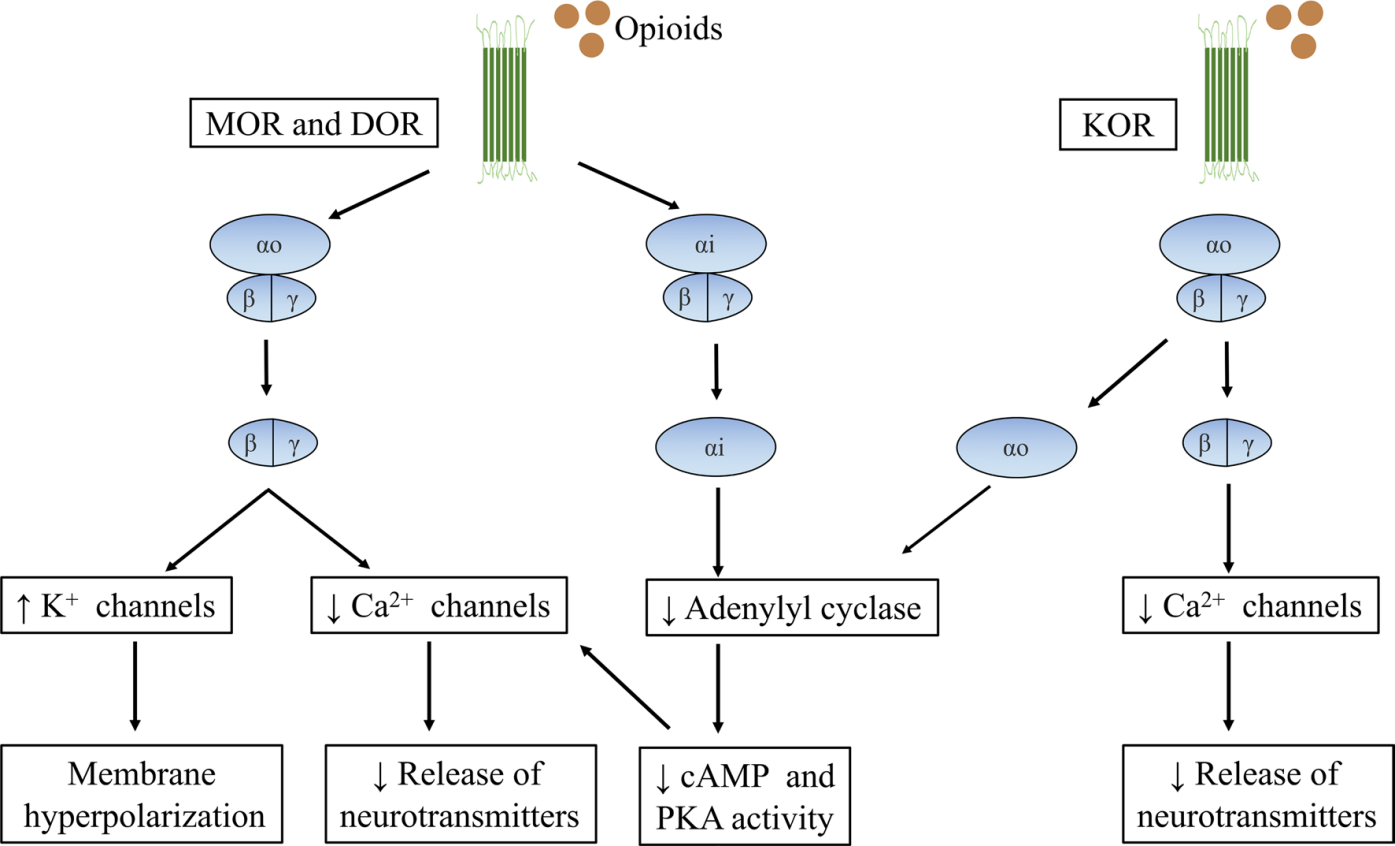
Activation of opioid receptors in the enteric nervous system. MOR mu opioid receptor, DOR delta opioid receptor, KOR kappa opioid receptor, cAMP cyclic adenosine monophosphate, PKA protein kinase A

Opioid receptors are widely distributed in the enteric nervous system (ENS) of the GI tract (Fig. 1). The primary opioid receptors include the mu opioid receptor (MOR), delta opioid receptor (DOR) and kappa opioid receptor (KOR). Opioid receptors located in the interneurons, secretomotor neurons and musculomotor neurons of the ENS mainly sustain the homeostasis of the GI tract [23]. Inhibition of neuronal excitability and imbalance of neurotransmitter release are the principal mechanisms by which opioids regulate the GI tract [24]. The activation of opioid receptors can change the concentration of K^+^ and Ca^2^^+^ by G protein-coupled receptors and then lead to the suppression of neuronal depolarization, decreased neuronal excitability and the inhibition of neurotransmitter release (Fig. 2) [25]. Circular muscle and longitudinal muscle in the GI tract have different nerve inputs, and opioid receptor agonists impair the normal motility of the GI tract by increasing the contraction activities of circular muscle and decreasing the contraction activities of longitudinal muscle through the suppression of neuronal excitability [26]. The inhibition of the excitatory neurotransmitters acetylcholine and substance-P participate in the discoordination motility of the GI tract, and the decrease of the inhibitory neurotransmitters vasoactive intestinal peptide and nitric oxide participate in the abnormal secretion and absorption of the GI tract [27].

In humans, MOR is expressed in both the myenteric and submucosal plexuses throughout the small and large intestine, and DOR and KOR are distributed in excitatory and inhibitory motor neurons in the human colon [28]. MOR is more distributed in the submucosal plexus than in the myenteric plexus. The activation of MOR can delay gastric emptying, increase gastrointestinal transit time, and suppress the secretion of water and electrolytes in the intestine. KOR appears more in the myenteric plexus. Most enteric neurons coexpress MOR and DOR receptors [29]. MOR and DOR are the primary opioid receptors for the regulation of enteric neurons. MOR and DOR are both distributed in the submucosal plexus and myenteric plexus, and the activation of MOR and DOR suppresses the excitation of enteric neurons by hyperpolarizing them, delays gastric emptying, increases the time of gastrointestinal transit, and suppresses the secretion of water and electrolytes in the intestine. The activation of KOR also inhibits the ENS contractile response, but its inhibition of the GI tract is weaker than that of MOR and DOR [30, 31].

## Methods

To investigate the effect of opioids on the GI function in the ICU, we searched PubMed and Embase from inception until September 2021. We used the keywords “ICU OR intensive care OR critically ill OR critical care”, “gastrointestinal OR gut OR constipation OR abdominal OR gastr* OR bowel”, “opioid* OR opiate*” and “clinical stud*”. Clinical studies associated with opioids and GI function in ICU adult patients were included. Paper in a language other than English and pediatric studies were excluded.

## Results

The initial search yielded 1155, with 520 in PubMed and 635 in Embase. After screening by title and abstract, 1103 were removed for duplication articles, reviews, pediatric studies, and articles that did not report the relationship between opioids and GI function in critically ill patients. 52 papers were screened by reading full articles. In the final, 26 articles reported the effect of opioids on the GI function in ICU patients, and one clinical study was added after reference search. The detailed information about the studies is listed in Table1, 2, and 4.

**Table 1:** Studies about the effect of opioids on gastric emptying in the ICU

Clinical trials	Study design	Population	Group	Study goal	Results	Type of opioids in the studies	The effect of opioids
McArthur et al. [38]	RCT	21 brain injured patients	M&M group (n = 11) vs propofol group (n = 10)	To compare the effect of opioids and nonopioid sedation on gastric emptying	Gastric emptying was no difference in brain injured patients between two groups	Morphine	Gastric emptying was not improved by avoiding morphine
Heyland et al. [32]	PCS	84 individuals	Mechanically ventilated patients (n = 72) vs healthy volunteers (n = 12)	To investigate variables that associated with impaired gastric emptying	Variables included age, sex and use of opioids	Morphine and morphine equivalent	Use of opioids and dose of opioids were associated with impaired gastric emptying
Bosscha et al. [36]	POS	16 individuals in Surgical ICU	Mechanically ventilated patients (n = 7) vs healthy volunteers (n = 9)	To determine the GI motility characteristics that associated with gastric intention	Morphine administration affected antroduodenal motility	Morphine	Opioids impaired the GI motility in mechanically ventilated patients
Mentec et al. [35]	POS	153 patients with nasogastric tube feeding	Normal GAV (n = 104) vs increased GAV (n = 49)	To investigate risk factors for increased GAV and UDI and complications	Risk factors: sedation, catecholamines Complications: higher incidence of pneumonia, longer ICU stay, higher mortality	Fentanyl	Opioids were risk factors for increased GAV
Nguyen et al. [37]	Descriptive study	36 individuals in mixed medical and surgical ICU	M&M group (n = 20) vs propofol group (n = 16)	To evaluate the effects of sedation with M&M vs propofol on gastric emptying	Patients receiving M&M had more impaired gastric emptying	Morphine	M&M inhibited gastric emptying
Berger et al. [82]	PS	105 patients in surgical ICU	NA	To assess the rate of the migration of the self-propelled feeding tube in the ICU patients	Self reported tubes can be considered as a promising tool to facilitate enteral nutrition	Morphine and fentanyl	Morphine was associated with lower progression rates
Chapman et al. [83]	POS	39 patients in the mixed medical and surgical ICU	Mechanically ventilated patients (n = 25) vs healthy volunteers (n = 14)	To explore the prevalence of delayed GE in the ICU and the relationships between scintigraphy and carbon breath test in the GE measurement	GE occurred in approximately 50% of critically ill patients, breath tests and scintigraphy both were valid method of GE measurement	Morphine	Administration of morphine was not associated with delayed GE

*RCT* randomized control study, *POS* prospective observational study, *PCS* prospective cohort study, *GAV* gastric aspirate volume, *UDI* upper digestive intolerance, *vs* versus, *M&M* morphine and midazolam, *GI* gastrointestinal, *PS* prospective study, *GE* gastric emptying, *NA* not applicable

**Table 2:** Studies about the effect of opioids on lower GI dysmotility in the ICU

Clinical trials	Study design	Population	Group	Study goal	Results	Type of opioids	The effect of opioids
Mostafa et al. [11]	POS	48 patients in the mixed medical and surgical ICU	Not constipated (n = 8) vs constipated (n = 40)	Constipation and its implications in the ICU	Delayed weaning from mechanical ventilation and enteral feeding	Alfentanil	Opioids had little effect on constipation
Van et al. [43]	Descriptive cohort study	44 individuals in mixed surgical and medical ICU	SDD (n = 22) vs control (n = 22)	To describe the influence of severity of illness, medication and selective decontamination on defecation	Severity of illness, vasoactive medication, morphine, duration of mechanical ventilation and length of ICU stay influenced the time to first defecate	Morphine	Morphine administration at least 4 days may be associated with delayed defecation
Nassar et al. [45]	POS	106 patients in the surgical ICU	Not constipated (n = 33) vs constipated (n = 73)	To determine the risk factors of constipation and its implications	Early enteral nutrition was associated with less constipation. Constipation was not associated with higher ICU mortality, length of stay and days free from mechanical ventilation	Fentanyl	Opioids were not associated with an increased incidence of constipation
Gacouin et al. [46]	POS	609 patients with mechanical ventilation at least 6 days	Late defecation (defecation ≥ 6 days after admission to ICU) group (n = 353) vs early defecation group (n = 256)	To determine the risk factors of late defecation and its implications	PaO_2_/FIO_2_ ratio of < 150 mm Hg and systolic blood pressure of < 90 mm Hg during the first 5 days of mechanical ventilation were independently associated with delayed defecation	Morphine	Unadjusted univariate analysis suggested that the use of opiates had an impact on the late defecation
Deane et al. [42]	POS	44 individuals mixed medical and surgical	Critically ill patients (n = 28) vs healthy volunteers (n = 16)	To determine small intestinal glucose absorption and small intestinal transit in critically ill patients	Critical illness was associated with reduced small intestinal glucose absorption	Not reported	Small transit were delayed in critical illness
Fukuda et al. 2016[47]	RS	282 patients who stayed in the ICU at least 7 days	Late defecation (defecation ≥ 6 days after admission to ICU) group (n = 96) vs early defecation group (n = 186)	To investigate the risk factors for late defecation and its association with the outcomes of ICU patients	Late enteral nutrition, sedatives and surgery were risk factors for late defecation, and late defecation was associated with a prolonged ICU stay	Fentanyl	Fentanyl was not a risk factor for late defecation
Prat et al. [48]	POS	189 patients	Not constipated (n = 91) vs all constipated (n = 98)	To determine the frequency and significance of constipation according to its definition criterion	Without laxation at least 6 days was more associated with specific outcomes	Sufentanyl	Opioids were associated with patients who constipated more than 6 days
Launey et al. [44]	POS	396 adults with at least 2 days’ invasive ventilation	NA	To determine the factors associated with the time to defecation	Non-invasive ventilation and the duration of ventilation were associated with the time to defecation	Morphine equivalents	Opioids were not associated with the time to defecation
Nguyen et al. [84]	POS	248 Mechanically ventilated patients receiving enteral nutrition	Patients with IGT (n = 50) vs patients without IGT (n = 198)	To determine the proportion and risk factors of critically ill adults with IGT	Pragmatically defined IGT was common in critical illness and associated with significant morbidity	Not reported	Use of opioids was identified as a risk factor for IGT

*RS* retrospective study, *POS* prospective observational study, *SDD* selective decontamination, *vs* versus, *IGT* impaired gastrointestinal transit, *NA* not applicable

### Upper GI dysmotility

Upper GI dysfunction occurs frequently in ICU patients [32]. Upper GI disorders include delayed gastric emptying, increased gastroesophageal reflux and abnormal duodenal contractions. All of these disorders increase the risk of nosocomial pneumonia [33]. A retrospective study describing the gut function of current ICU patients indicated that gastric emptying was abnormal, and most enterally fed patients exhibited large gastric aspirates [34]. Delayed gastric emptying is one manifestation of feed intolerance and can result in inadequate nutrition. Sedation with the combination of midazolam and fentanyl was an independent factor for increased gastric aspirate volume and upper digestive intolerance for enteral nutrition [35]. Opioids inhibit gastric emptying in a dose-dependent pattern [32]. A prospective cohort study measured the variables associated with impaired gastric emptying using the acetaminophen absorption model [32]. Before acetaminophen treatment, 24 patients were infused with morphine or a morphine equivalent; patients treated with opioids took longer to reach maximum concentration of acetaminophen, and a high dose of opioids accompanied significant impairment of gastric emptying. A study analysed the effect of opioids on GI motility in patients on mechanical ventilation [36]. Compared with nine healthy volunteers, the migrating motor complex (MMC) of seven mechanically ventilated patients treated with morphine was significantly shortened. Meanwhile, the gastric retention of the patients was more than 20% due to the abnormal motility pattern of the MMC. When morphine was stopped, the motility pattern of the stomach could convert to a normal pattern, and mean gastric retention was decreased [36]. Nguyen et al. evaluated the effect of morphine and midazolam (M&M) or propofol on gastric emptying by gastric scintigraphy [37]. The half-life of gastric emptying in patients receiving M&M was 153 min, longer than the half-life of 58 min in the control group receiving propofol, and M&M-treated patients had higher proximal meal retention because morphine inhibited gastric emptying by increasing retrograde duodenal contractions and pyloric tone.

Only a prospective randomized study compared the effect of morphine plus midazolam and propofol sedation on 21 ventilated patients with brain injuries by the paracetamol absorption mode [38]. However, there was no difference between the two groups because the study had a small sample size to detect a difference in gastric emptying, and gastric emptying was influenced by other factors that were more important than opioids [38]. Nguyen et al. analysed six cohort studies with 132 patients to explore gastric emptying in critically ill patients. The authors reported that opioid medication had no association with delayed gastric emptying. Even though the sample size of this study was larger than the others, it had limitations similar to retrospective studies [39]. Table 1 lists clinical studies about the effect of opioids on gastric emptying in the ICU. The results regarding opioids in gastric emptying were inconsistent, and only a randomized study and a pooled analysis suggested that opioids had little effect on gastric emptying. Other studies demonstrated that opioids increased the risk for impaired gastric emptying, but these studies were mostly observational studies with relatively low-quality evidence, and none of these studies excluded the effects of sedatives, such as propofol and midazolam. Sedatives also had an inhibitory effect on GI function. The GI function of critically ill patients is vulnerable and can be affected by many other factors. The exact role of opioid administration in delayed gastric emptying is unclear in the ICU setting due to sparse data.

### Lower GI dysmotility

The inhibition by opioids in lower GI transit has been described in detail [21, 40, 41]. However, studies of their effects on the small intestines of critically ill patients are rare. A prospective study demonstrated that opioids inhibited the small intestinal transit of ICU patients [42]. Constipation is a common presentation of lower gastrointestinal dysmotility in ICU patients. However, the definition of constipation in critically ill patients is not agreed on [43]. The association between the clinical outcomes of ICU patients and the time of defecation is controversial. Some studies have suggested that constipation was associated with increased mortality, but some studies demonstrated that constipation was related to a more prolonged ICU stay but not mortality [44–46]. Therefore, it should be recognized that it is not whether defecation impacts mortality but failure to defecate after medication [45].

Opioids are an important factor in the development of constipation [34]. Constipation occurred in 16–83% of patients in the ICU and was associated with delayed enteral feeding, bacterial translocation, and delayed weaning from mechanical ventilation [11]. However, there is no direct evidence that opioid administration is independently associated with time to defecate in critically ill patients [44]. Some studies only indirectly demonstrated that opioid administration had little effect on constipation in the ICU setting (Table 2).

**Fig. 3:**
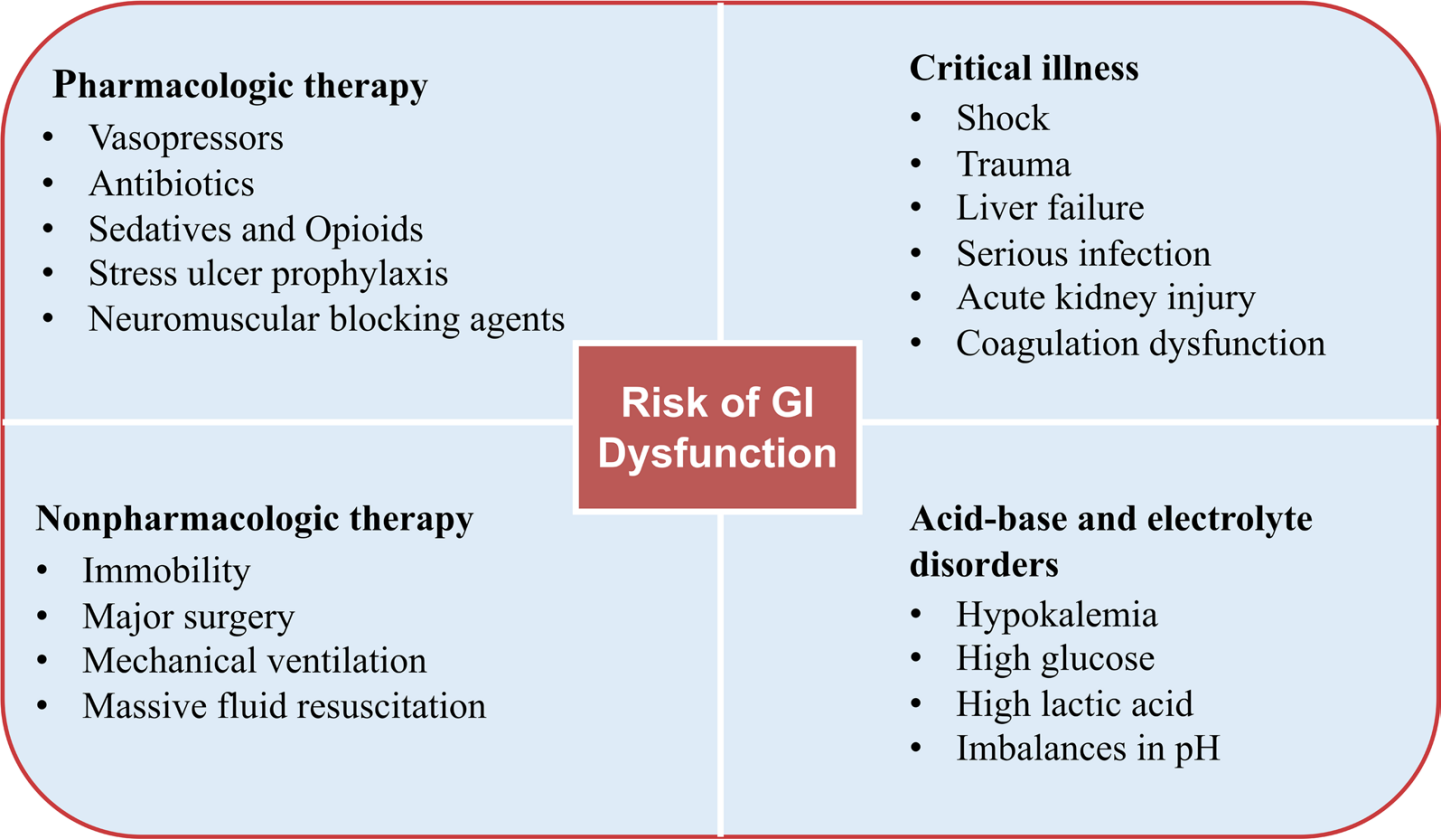
Risks of GI dysfunction in the ICU

In a prospective study of motility of the lower GI tract in ICU patients, the authors found similar use of alfentanil in patients with and without constipation, which suggested that alfentanil had little effect on constipation. The authors also suggested that constipation was not associated with mortality in this study [11]. The administration of fentanyl in ICU patients was also not associated with an increased risk of constipation [45, 47]. However, a descriptive cohort study demonstrated that prolonged treatment with morphine was a risk factor for late defecation in critically ill patients, but morphine administration was significantly different between early defecation and late defecation on Days 5, 6 and 7 in the ICU, but not in the first 4 days [43]. In this study, late defecation was defined as defecation six days after admission to the ICU [43]. Dominique et al. also proposed that the use of sufentanyl and midazolam were risk factors for patients’ constipation between 3 and 6 days [48]. All these studies did not directly compare the effect of opioid administration on constipation, and different opioid drugs and the duration of opioid administration may have different effects on GI function. The effect of opioids on GI function depends on detailed opioid administration and infusion time. The long-term use of morphine in patients might reflect a more critical illness and multiple therapies; therefore, it is difficult to distinguish the effect of morphine or the illness itself and other factors on GI dysfunction [43]. GI motility is sensitive to various stresses and critical illnesses (Fig. 3), and critical illness itself can also cause GI dysmotility by diminished numbers of interstitial cells of Cajal in the GI tract [49]. Therefore, whether opioid administration delayed defecation in ICU patients is unclear.

### Potential increased infection risk by opioids

The microbiome is one of the crucial parts of the GI barrier. However, the composition and diversity of the microbial community are severely impaired in critically ill patients and can further exacerbate illness progression [50]. Opioids are increasingly considered to cause immunosuppression, compromise the GI barrier, alter microbiome function and increase infection risk [51]. Disturbances in microbiological composition and diversity were associated with the mortality of critically ill patients [52]. Opioids can expedite the invasion of the pathogen to the host and increase the mortality of septic mice [53]. Pathogen clearance is impaired in septic animals treated with morphine, resulting in a higher bacterial load in different organs [53]. Opioids can also enhance susceptibility to Pseudomonas aeruginosa, Enterococcus faecalis, and Listeria monocytogenes [53–57]. Both morphine and methadone administration can lead to high mortality in septic mice, and morphine contributes to bacterial dissemination and overproduction of the proinflammatory cytokine interleukin-17A (IL-17A) through activation of TLR2 and disrupts the IL-23/IL-17-mediated defence system of innate and acquired immunity [53, 58]. The overexpression of IL-17A plus morphine treatment can increase GI permeability, impair gut epithelial barrier function and lead to higher bacterial load and higher mortality [53]. Morphine treatment increases Citrobacter rodentium virulence and dissemination into the mesenteric lymph nodes, spleen, liver and blood, promotes bacterial adherence to the small intestine, and disrupts the integrity of the epithelial barrier and the IL-17A immune response. [52]. Citrobacter rodentium alone cannot contribute to severe GI damage in the early phase of infection [52]. Morphine-induced changes in the GI microbiome occur in a receptor-dependent manner and can be inhibited by opioid antagonists [51, 55]. The impact of morphine administration on pathogen virulence has been well studied, but the effect of other opioids on virulence is unclear [59].

Some clinical studies have suggested that opioids can destroy the immune response and increase susceptibility to infection [60]. Several cohort studies have demonstrated that opioid treatment leads to taxonomic changes and changes in richness and diversity in the gut microbiota [61–63]. A retrospective cohort study also found that patients using long-acting opioids with previously reported immunosuppression had the greatest risk of serious infection compared to patients without previously reported immunosuppression [60]. Many critically ill patients are immunocompromised, and it is essential to carefully select opioid drugs to avoid promoting further immunosuppression [64]. The serum level of morphine was found to be dramatically increased in patients with severe sepsis or septic shock [65]. A retrospective survey illustrated the impact of opioid analgesics on postburn infection complications. High opioid intake was associated with infectious complications in patients with mild to moderate (< 26% total body surface area) injuries [66]. However, studies about potential infection risks of opioids are rare in the ICU setting: more evidence is needed for critically ill patients.

### Treatment of GI dysmotility with opioid antagonists

Although patients are traditionally given an osmotic laxative or a stimulant laxative to treat constipation, constipation still causes serious GI complications such as bowel perforation and even death [67]. Opioid antagonists are often a last-line medication for patients with opioid-induced constipation (OIC) in the ICU [67]. Opioid antagonists can be divided into peripheral acting or both peripheral-mediated and central-mediated antagonists (Table 3). Peripherally acting mu opioid receptor antagonists (PAMORAs) have a limited ability to breach the blood–brain barrier, hence having no effect on the analgesic benefits, and have a blunt effect of opioids in the GI system [68]. Centrally mediated opioid antagonists can ameliorate GI hypomotility but may cross the blood–brain barrier and reverse analgesia [69].

**Table 3:** Information about opioid antagonists

Opioids antagonists	Mechanism of action	Administration	Recommended dose	Approved indication	Side effects	Contraindications
Methylnaltrexone	PAMORA	Subcutaneous injection Oral	8 mg (BW 38–62 kg) 12 mg (BW 63–114 kg) 0.15 mg/kg for patients with weights outside of these ranges	OIC patients, lower GI paralysis, insufficient response to laxatives	GI Perforation abdominal pain, nausea, diarrhea, flatulence	Known or suspected mechanical GI obstruction, perforation
Alvimopan	PAMORA	Oral	12 mg BID (limited to 15 doses)	Postoperative ileus, partial bowel resection, primary anastomosis	MI, anemia, dyspepsia, hypokalemia, back pain, urinary retention	Opiate use > 1 week
Naloxegol	Antagonist of DOR, KOR and MOR	Oral	12.5–25 mg OD	OIC in non-cancer and chronic pain patients, inadequate response to laxative therapy	Nausea, vomiting, diarrhea, abdominal pain, and bowel obstruction, flatulence, MI and QT prolongation less than Alvimopan	Patients with known or suspected GI obstruction are at increased risk of perforation
Naloxone	Opioid receptor antagonist mediated both peripheral and central	Oral	3–12 mg TID	Constipation, lower GI paralysis	Symptoms of opioid withdrawal, reversal of analgesic	Hypersensitivity to the drug
Naldemedine	Peripherally acting DOR, KOR and MOR antagonist	Oral	0.2 mg OD	OIC patients	Abdominal pain, diarrhea, nausea, gastroenteritis	Suspected or known GI perforation, obstruction and severe hepatic disease

*PAMORA* peripherally acting mu opioid receptor, *MOR* mu opioid receptor, *DOR* delta opioid receptor, *KOR* kappa opioid receptor, *BID* twice per day, *TID* three times per day, *BW* body weight, *OD* once per day, *MI* myocardial infarction, *OIC* opioid-induced constipation

### Methylnaltrexone

Methylnaltrexone is a methylated form of naltrexone that can be administered as an oral formulation and a subcutaneous injection [70]. Methylnaltrexone has been demonstrated to be safe and beneficial for the treatment of OIC in both preclinical studies and clinical studies. In mice, methylnaltrexone can effectively reverse morphine-induced reductions in the inhibition of bowel contraction in a concentration-related manner when administered 15 min before morphine and without compromising analgesia [71]. In guinea pigs, methylnaltrexone also reversed GI inhibition by chronic morphine treatment, but methylnaltrexone had no effect on GI transit without opioid stimulation [72]. In humans, methylnaltrexone was first approved by the Food and Drug Administration (FDA) for the treatment of OIC in patients with advanced illness when the response to laxative therapy was insufficient [68]. However, studies on the safety of methylnaltrexone in the ICU is rare. The methylnaltrexone for the Treatment of Opioid Induced Constipation and Gastrointestinal Stasis in Intensive Care Patients (MOTION) trial was a multicentre, double-blind, randomized placebo-controlled trial. This study aimed to investigate whether methylnaltrexone alleviated OIC in critical care patients [73]. The MOTION trial enrolled 84 patients; 41 patients in the methylnaltrexone group and 43 in the placebo group were finally analysed. However, this study found no significant difference in time to rescue-free laxation (Hazard ratio 1.42, 95% CI 0.82–2.46, *p* = 0.22) or in gastric residual volume between the groups.

### Naloxone

Naloxone is mu-opioid antagonist approved by FDA for the reversal of respiratory depression caused by opioid overdose. Naloxone alleviated the inhibitory effects of the central and peripheral opioid systems on the whole and upper GI transit in mice [74]. However, naloxone can cause centrally mediated jumping behaviour in animals [75]. Several clinical studies have investigated the effect of naloxone in the ICU setting. A prospective, randomized, double-blinded study evaluated the effect of enteral naloxone on the amount of gastric tube reflux and the time of first defecation in mechanically ventilated patients who received fentanyl analgesia [76]. This study provided evidence that the administration of enteral opioid antagonists in ventilated patients reduced gastric tube reflux, but there was no difference in the time of first defecation. Naloxone did not contribute to changes in the sedation score, vital signs, fentanyl dose, midazolam dose or propofol dose in the ICU setting and appeared to be safe for OIC treatment in the medical intensive care unit (MICU) [77, 78]. However, a double-blind, randomized, placebo-controlled study suggested that nine patients who received oral naloxone had enhanced bowel frequency, and three of the nine patients had reversal of analgesia [79]. The study showed that patients treated with higher doses of opioids appeared to be more sensitive to the analgesic reversal effect of oral naloxone, and even dividing the dose could cause the reversal of analgesia [67, 79].

### Alvimopan

Alvimopan, a PAMORA, was approved by the FDA to accelerate GI function after partial large- or small-bowel resection surgery with primary anastomosis [68]. Alvimopan can reverse the delayed GI transit resulting from intestinal manipulation without the administration of opioids before surgery and improve postoperative ileus accompanied by morphine administration in a rat model of ileus [80]. Intestinal manipulation can upregulate the endogenous opioid pathway and increase the release of endogenous opioid peptides [80]. The reversal of alvimopan for delayed GI transit may be mediated via inhibition of endogenous opioid release. There are currently no clinical studies about alvimopan use in the ICU setting.

### Contradicted results about the use of opioid antagonists in the ICU

At present, the most studied opioid antagonists in the ICU are methylnaltrexone and naloxone. Studies on methylnaltrexone and naloxone in the ICU setting are insufficient, and the results of their roles in the recovery of GI motility are contradictory. Most studies have suggested that naloxone and methylnaltrexone appeared to be effective and safe in treating OIC, but two RCT studies concluded that both of the medications had little effects for OIC treatment in the ICU [73, 76]. A possible reason for this outcome may be the study design. The RCTs matched baseline between groups, the distribution of confounding factors was also well balanced and there was a small bias compared with the retrospective study. Another reason is the number of patients. Studies with a smaller number of patients have a smaller fragility index, and several incidental events can reverse the study results. Meanwhile, a retrospective review assessed the effectiveness and safety of naloxone and subcutaneous methylnaltrexone for OIC treatment in the MICU. This single-centre study included 100 patients who received continuous fentanyl infusions for at least 72 h, and the primary outcome was the time to first bowel movement. The results showed no difference in the primary outcomes, and the median times to first bowel movement for naloxone and subcutaneous methylnaltrexone were 30 and 24 h, respectively (*P* = 0.165) [81]. It is difficult to compare studies of different opioid antagonists because of highly heterogeneous endpoints. There is no definitive conclusion about the effect of opioid antagonists on GI function in critically ill patients because of the relatively low quality of evidence and insufficient data in the ICU. Table 4 lists clinical studies associated with opioid antagonists in the ICU.

**Table 4:** Studies about opioid antagonists in the ICU

Clinical trials	Study design	Population	Group	Study goal	Conclusion
Liu et al. [79]	RCT	9 OIC patients	NTX (n = 6) vs placebo (n = 3)	To evaluate the effect of low doses of NTX on constipation and analgesia	Bowel frequency improved but reversal of analgesia also occurred
Meissner et al. [76]	RCT	84 mechanically ventilated patients received intravenous infusion of fentanyl	NTX (n = 38) vs placebo (n = 43)	To study the effect of NTX on the gastric tube reflux, the occurrence of pneumonia, and the time to first defecation	The administration of NTX was effective to reduce gastric tube reflux and frequency of pneumonia, but the first defecation in the two groups did not differ
Arpino et al. [78]	RS	26 OIC patients in the ICU received opioids at least 48 h prior to NTX	Comparison between before and after NTX administration	To assess the safety of NTX in the ICU	The administration of NTX was not associated with changes in sedation score, vital signs, fentanyl dose, midazolam dose or propofol dose
Gibson et al. [77]	RS	16 OIC patients received scheduled doses of opioids for pain control in the MICU	Before and after during NTX treatment	To evaluate the efficacy and safety of NTX before and after during naloxone treatment	NTX appeared to be effective and safe for the treatment of OIC in the MICU. NTX had no reversal of analgesic
Merchan et al. [81]	RS	100 OIC patients in the MICU	NTX (n = 52) vs MNTX (n = 48)	To assess the effectiveness and safety of NTX and MNTX for the treatment of OIC	MNTX and NTX appear to be effective and safe for the treatment of OIC. The median times to first BM for NTX and MNTX were 30 and 24 h (*P* = 0.165)
Patel et al. [73]	RCT	84 OIC patients in the ICU with opioid analgesics for at least 24 h	MNTX (n = 41) vs placebo (n = 43)	To investigate whether MNTX alleviates OIC in critically ill patients	There was no evidence to support the addition of MNTX to regular laxatives for the treatment of OIC in the ICU
Sawh et al. [85]	RS	15 OIC patients in the ICU	MNTX (n = 7) vs conventional rescue therapy (n = 8) to treat the OIC in the ICU	To compare the effect of MNTX and conventional rescue therapy in the ICU	MNTX was effective in the treatment of OIC patients
Masding et al. [86]	ROS	827 patients in the cardiothoracic ICU	NA	the prevalence of OIC in a large cardiothoracic ICU and the use of naloxegol	Nearly 20% of ICU patients had OIC and use of naloxegol could reduce the prevalence of OIC
Phase 3 [87]	RCT	350 ICU patients received opioids at least 48 h hours	Polyethylene glycol vs naloxegol	To investigate whether naloxegol is superior to osmotic laxatives for refractory constipation in ICU patients	The study had withdrawn

*vs* versus, *RCT* randomized control study, *RS* retrospective study, *OIC* opioid-induced constipation, *MICU* medical intensive care unit, *NTX* enteral naloxone, *MNTX* methylnaltrexone, *BM* bowel movement, *NA* not applicable

## Future directions and research

GI function is strongly correlated with the clinical outcome of ICU patients. It is important to identify and avoid potential risk factor s for GI injury in clinical work. Opioids are frequently administered to treat pain and have potential risks for the impairment of GI function. Therefore, the role of opioids in GI dysfunction in critically ill patients should be clarified for precision medicine. What is the extent to which opioids affect GI function in the ICU setting, and what is the proportion of opioids in the causative factors of AGI in the ICU setting? Whether different types, doses and duration of opioid administration have different impacts on GI function remains unclear in the ICU. What is the best choice of opioids for critically ill patients with AGI and higher risks of AGI? Finally, the most commonly used GI motility drugs are laxatives, metoclopramide and erythromycin in the ICU setting. Patients who have an inadequate response to these drugs have great potential to receive operative treatment. In addition, most of these patients who received operative treatment have a poor prognosis. Therefore, it is imperative to evaluate the effectiveness and safety of other GI motility drugs in the ICU setting, such as opioid antagonists.

## Conclusion

In conclusion, pain is common in the ICU; more than half of ICU patients suffer moderate to serious pain, and it is necessary to use analgesia. Opioids have a considerable role in pain management in the ICU setting, and various studies have demonstrated the inhibition of GI motility by opioids. However, the GI tract is highly vulnerable in critically ill patients, and many factors can influence GI function from the GI barrier and absorption to motility in the ICU setting. The extent to which opioids contribute to GI dysmotility is unclear in the ICU setting due to the paucity of published data. GI dysmotility is on the severe side of the spectrum and should be adequately managed. However, at present, medication for GI dysmotility is limited in the ICU setting. Therefore, it is crucial to maintain a good balance between GI function and opioid administration. In the prescription of opioid drugs, many properties need to be taken into account before medication choices are finalized.

## Data Availability

Not applicable.

## Abbreviations

GI: gastrointestinal
WGAP: Working Group on Abdominal Problems
AGI: acute gastrointestinal injury
ENS: enteric nervous system
MOR: mu opioid receptor
DOR: delta opioid receptor
KOR: kappa opioid receptor
MMC: migrating motor complex
M&M: morphine and midazolam
IL-17A: interleukin-17A
OIC: opioid-induced constipation
PAMORA: peripherally acting mu opioid receptor antagonists
MOTION: the methylnaltrexone for the treatment of opioid induced constipation and gastrointestinal stasis in intensive care patients
MICU: medical intensive care unit
FDA: food and drug administration
